# *De Novo* sequencing and transcriptome analysis for *Tetramorium bicarinatum*: a comprehensive venom gland transcriptome analysis from an ant species

**DOI:** 10.1186/1471-2164-15-987

**Published:** 2014-11-18

**Authors:** Wafa Bouzid, Marion Verdenaud, Christophe Klopp, Frédéric Ducancel, Céline Noirot, Angélique Vétillard

**Affiliations:** Venoms and Biological Activities Laboratory, EA 4357, PRES-University of Toulouse, Jean-François Champollion University Center, Albi, France; Department of Pharmacology and Immunoanalysis, CEA, iBiTec-S, Gif-sur-Yvette, F-91191 France; The GenoToul bioinformatics platform, UR875 Biométrie et Intelligence Artificielle, INRA, Castanet-Tolosan, 31326 France

**Keywords:** *Tetramorium bicarinatum*, Social hymenoptera, Ant, Venom glands, Venom toxins, Hymenopteran allergens, *de novo* assembly, New generation sequencing, Illumina technology

## Abstract

**Background:**

Arthropod venoms are invaluable sources of bioactive substances with biotechnological application. The limited availability of some venoms, such as those from ants, has restricted the knowledge about the composition and the potential that these biomolecules could represent. In order to provide a global insight on the transcripts expressed in the venom gland of the Brazilian ant species *Tetramorium bicarinatum* and to unveil the potential of its products, high-throughput approach using Illumina technology has been applied to analyze the genes expressed in active venom glands of this ant species.

**Results:**

A total of 212,371,758 pairs of quality-filtered, 100-base-pair Illumina reads were obtained. The *de novo* assemblies yielded 36,042 contigs for which 27,873 have at least one predicted ORF among which 59.77% produce significant hits in the available databases. The investigation of the reads mapping toxin class revealed a high diversification with the major part consistent with the classical hymenopteran venom protein signature represented by venom allergen (33.3%), followed by a diverse toxin-expression profile including several distinct isoforms of phospholipase A_1_ and A_2_, venom serine protease, hyaluronidase, protease inhibitor and secapin. Moreover, our results revealed for the first time the presence of toxin-like peptides that have been previously identified from unrelated venomous animals such as waprin-like (snakes) and agatoxins (spiders and conus).

The non-toxin transcripts were mainly represented by contigs involved in protein folding and translation, consistent with the protein-secretory function of the venom gland tissue. Finally, about 40% of the generated contigs have no hits in the databases with 25% of the predicted peptides bearing signal peptide emphasizing the potential of the investigation of these sequences as source of new molecules. Among these contigs, six putative novel peptides that show homologies with previously identified antimicrobial peptides were identified.

**Conclusions:**

To the best of our knowledge, this work reports the first large-scale analysis of genes transcribed by the venomous gland of the ant species *T. bicarinatum* and helps with the identification of Hymenoptera toxin arsenal. In addition, results from this study demonstrate that *de novo* transcriptome assembly allows useful venom gene expression analysis in a species lacking a genome sequence database.

**Electronic supplementary material:**

The online version of this article (doi:10.1186/1471-2164-15-987) contains supplementary material, which is available to authorized users.

## Background

The biodiversity and specificity of animal venom compounds make them an uncommon and invaluable source from which pharmacological and therapeutic agents can be established [1]. In Hymenoptera, the venom gland shows plasticity associated with organism life history diversification and venom compounds have evolved as important weapons used for colony or individual defense [2]. Unlike other venoms such as from snakes, hymenopteran stings are generally not lethal causing mainly inflammatory and/or immunological reactions despite that some venoms from solitary Hymenoptera have evolved to cause paralysis to permit egg laying on their arthropod hosts [2]. Toxin peptides from social Hymenoptera follow similar general features with short and linear polycationic peptides, responsible of cell lysis, hemolysis, histamine release and antimicrobial actions [3, 4]. Current knowledge of venom proteins and peptides involved in these processes is rather limited to stinging model species from wasps, bees, ants [5] or solitary endoparasitoid wasps [6]. Hence, little data are available from other groups especially from ants in spite of the astonishing taxonomic diversification in this insect group [5, 7]. Unlike stings from social bees and wasps that are solely used for defense, those from ants have additional functions as prey capture, aggregation and social communication which implies more diverse venom compounds. In addition, composition of venoms from ants varies significantly between the different ant subfamilies with probably unique venom components specific to each group which warrants their investigation [7]. This fact has been approved until recently by a comprehensive study of the venom gland transcriptome of the giant ant *Dinoponera quadriceps* that revealed species-specific toxin diversification [8].

In addition to biochemical investigation, ‘Venomics’ involving cutting-edge transcriptomics, proteomics and high-throughput venom peptide characterization technologies are emerging projects aiming at unraveling animal venom complexity for both fundamental and practical aspects [9, 10]. Given that venom proteins/peptides are produced in dedicated glands, transcriptome sequencing has proven to be an effective approach to identifying the expressed toxin genes. This is of particular interest for venoms that are difficult to sample such as mandibular venoms or for animals producing limited venom amounts. The latter case applies in particular to ants, which are, due to their tiny body and venom apparatus size, difficult to screen by analytical investigation unless laborious venom sac dissection is undergone with many thousands of individuals sacrificed [11].

Our study species *Tetramorium bicarinatum* [Hymenoptera: Formicidae] was chosen because of the diversity of its biotopes and also because this ant genus is more primitive than other highly derived genus (such as the massively studied genus *Solenopsis*). Hence, it is considered to produce more protein venom [12]. In a previous study, we generated Expressed Sequence Tags (ESTs) from the venom-gland transcriptome of this species, however the study relied on Sanger sequencing, generating important, but ultimately limited data [13]. For non-model organisms lacking defined genomes such as our studied species, *de novo* assembly is typically required for downstream RNA-Seq analyses [14].

In the current study, we characterized the transcriptional expression in the venom gland cells of the ant species *T. bicarinatum* using next generation illumina sequencing technology. Along with *de novo* assembly and transcriptome annotation, analysis of global patterns of gene expression and functional categorization were performed. In addition, features of some relevant putative toxin candidates are discussed.

## Results and discussion

### Illumina NGS and read assembly

**Figure 1:**
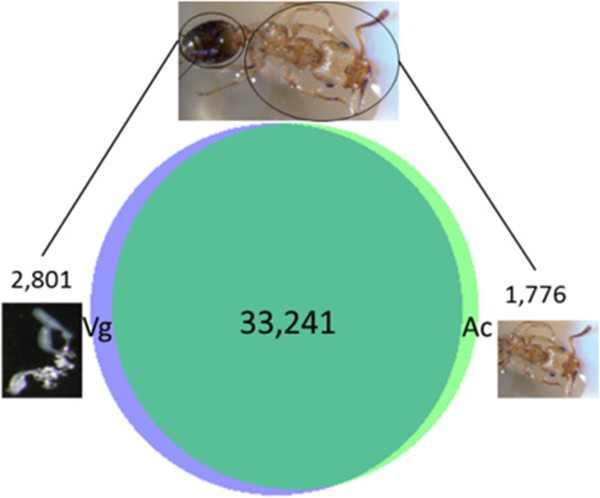
**Number of Illumina contigs generated by** ***de novo*** **assembly from** ***Tetramorium bicarinatum.*** Vg refers to venom glands and Ac to ant carcasses. 33,241 contigs were shared by both samples and 2,801 contigs have only been detected in venom glands.

**Figure 2:**
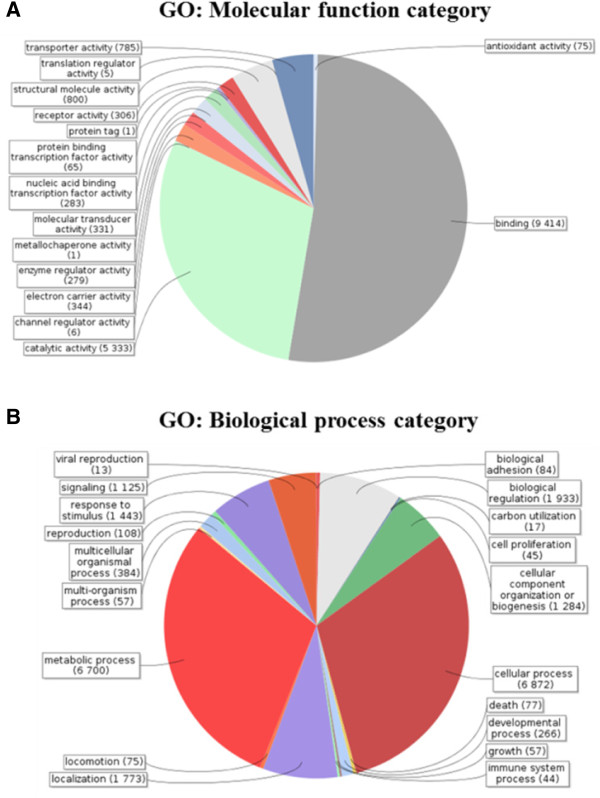
**Gene ontology (GO) functional categorization of the generated sequences from** ***T. bicarinatum*** **venom glands. A**: GO terms assigned in relation to the molecular function, **B**: GO terms assigned according to the biological process.

The gene expression profile of *Tetramorium bicarinatum* venom glands were deduced from mRNA samples of ant whole body and venom gland tissues using the Illumina sequencing approach. After the sequencing quality filtering step, a total of 424,743,516 of 100-base-pair Illumina reads were obtained for both samples (Table 1). The *de novo* assemblies yielded 37,818 contigs for the two libraries (venom glands and ant carcasses) among which 33,241 contigs were shared by both tissues (Figure 1). All unassembled sequencing reads, which accounted for 25% for the ant library and 50% for the venom gland library, were excluded from our investigation. As the aim of this study was to profile venom transcriptome, we mainly focus on the 36,042 contigs recovered from the venom gland library. The contigs recorded from the ant whole body sample were used to assess differentially expressed genes. Among these detected transcripts (contigs), 27,873 have at least one predicted ORF with 59.77% producing significant hits in the available databases. Gene ontology (GO) functional categorization of the annotated contigs from *T. bicarinatum* venom glands is provided on Figure 2. The GO analysis in relation to molecular functions revealed that the largest number of transcripts was related to protein binding, followed by catalytic activities and at a lower rate to structural protein and transporter activities (Figure 2A). Analysis of the biological processes revealed two dominant major gene categories of cellular and metabolic processes. The following categories are related to biological regulation, localization, response to stimulus, biogenesis and signaling (Figure 2B).

**Table 1:** **Summary of the** ***T. bicarinatum*** **trascriptome cDNA libraries from venom gland and the whole body tissues**

Total number of reads	424,743,516
Total base pairs	42,474,351,600
Average read length	100 bp
Total number of contigs	37,818
Number of contigs having mapped reads from ant library	35,017
Number of contigs having mapped reads from venom gland library	36,042
Number of unigenes from gland library (based on the annotation)	28,294
Mean length of contigs	1839 bp
Total number of sequences with at least one transcribed sequence	27,873

**Figure 3:**
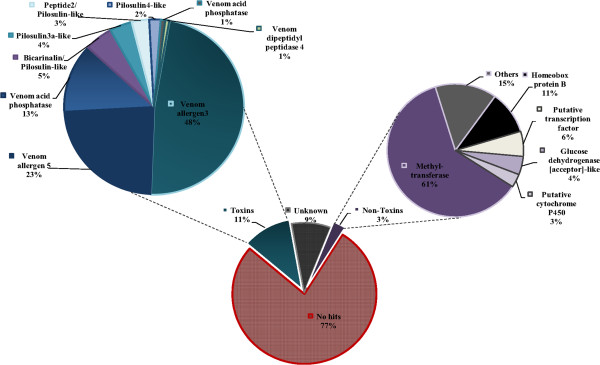
**Composition of the venom-gland ‘over-expressed’ protein/peptides predicted from the venom gland transcripts of** ***T. bicarinatum*** **.** Details on the composition of the toxin and non-toxin group are given. Unknown group (9%) designates the contigs that matched hypothetical or uncharacterized proteins.

In order to assess differentially expressed genes in the venom glands, we used the previously described *DESeq* approach implemented in R package which allows investigation of differential expressed transcripts from high-throughput data without replicates [15]. Regarding a likely important expression level variation between the two tested samples, the analysis failed to give workable results. This bias could be linked to the necessary pre-amplification of the venom gland sample prior to library construction compared to ant whole body sample from which extracted RNA quantity was sufficient to be directly processed to cDNA library construction (see Methods section). To bypass this bias probably originating from the amplification step, we set an arbitrary 10^3^ fold expression-threshold to the 37,818 contigs obtained from both sequenced samples. Due to a lower quality of the sequencing of the read2, we decided to perform our analysis using only the read1 for both samples. In total, we generated a list of 502 contigs that are 1000 fold more expressed in the venom gland, that we called the ‘venom-gland over-expressed’ transcripts. Analysis of the functional diversification among this group revealed a significant abundance (77%) of contigs that have no hits in the databases (Figure 3). On the other hand, contigs that matched predicted toxins and non-toxin proteins, accounted for 11% and 3%, respectively. The remaining group of 9% consisted of uncharacterized compounds (Figure 3). Moreover, about 235 contigs have significant hit with available bacterial sequences. These sequences have not been eliminated given that their low number does not affect the overall assemblage quality. The distribution of the possible bacterial community cohabiting with *T. bicarinatum* according to their contig number is provided in (Additional file 1: Figure S1).

### Cluster relevant to cellular functions

Among the 502 ‘over-expressed’ transcripts, 3% clusters presented significant hits in the databases involved in various cellular functions (Figure 3). The most relatively high expressed transcripts of this category are those matching methyl-transferases (61%) having transcription co-activator activities. This finding is in agreement with previous work showing that ‘gene transcription protein’ are the most abundant cellular transcripts reflecting the functional feature of the specialized tissues such as venom gland cells [16]. In addition, significant transcripts (4%) matched the glucose dehydrogenase involved in carbohydrate metabolism. This enzyme, that belongs to a family of oxido-reductases, has been identified in a recent work based on genome mining and proteomic analysis and was described as a novelty in insect venoms [6].

### Cluster related to venom toxins

Venoms from social Hymenoptera are important defensive weapons in which the most common components are low molecular proteins recognized as important allergens and resulting in an IgE-mediated reaction [17]. The investigation of the reads mapping to a toxin class that accounted for 11% of the ‘over-expressed’ genes revealed a high diversification with the major part represented by hymenopteran allergens (Additional file 2: Table S1). As can be seen in Figure 3, venom allergens 3 and 5 have been found among the main ‘over-expressed’ transcripts in the toxin-like group (48% and 23%, respectively). Venom allergen 3 (*Sol i* 3) described in the fire ant *Solenopsis invicta* is closely related to vespid venom antigen 5 and represents the major allergen of a series of *Sol i* peptides identified as the most frequent cause of hypersensitivity reactions following sting from this species [18]. Amino acid sequence comparison with related *Sol i* 3 allergens from our previous work shows special signature of *Sol i* 3-like from *T. bicarinatum* which would be relevant in the synthesis of recombinant venom allergens for immunotherapy [13]. In addition to venom allergen 3 and 5, transcripts encoding venom acid phosphatase were detected among the major expressed toxins/allergens (13% of the putative toxins, Figure 3). This enzyme has been found in relatively high levels in the honey bee and some social wasp venoms and recognized as potent releaser of histamine in human basophils [19, 20]. Its biological function in Hymenoptera is however still unclear but appears to have evolved as a deterrent for vertebrate predators [5].

**Figure 4:**
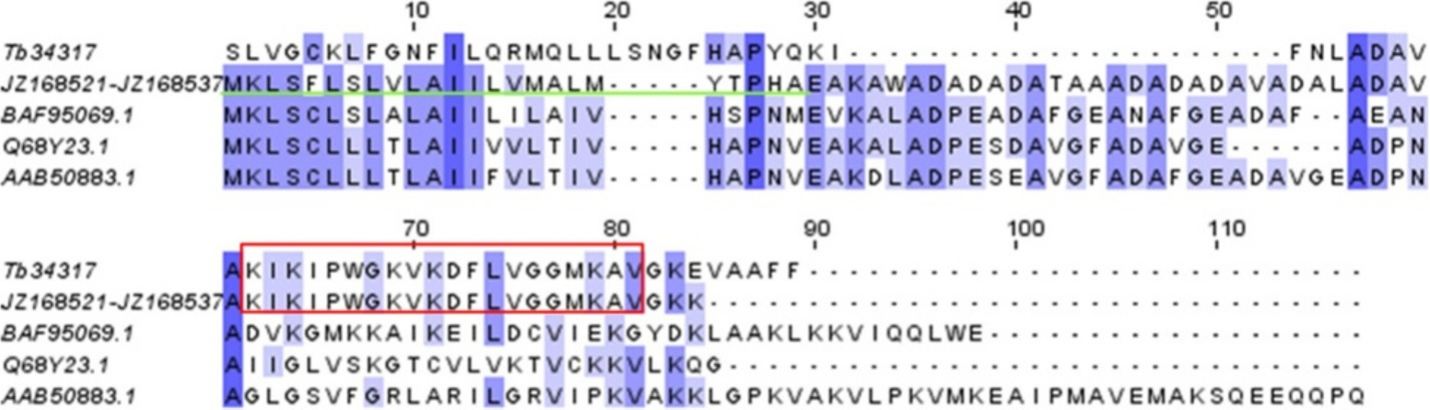
**Amino acid sequence alignment of representative of pilosulins and pilosulin-like from different ant species.** The sequence of bicarinalin/pilosulin-like (Tb34317) from *T. bicarinatum* was aligned with sequences of the pilosulin family from *Myrmecia banksi* [GenBank: Q68Y23.1 and BAF95069] and *Myrmecia pilosula* [GenBank: AAB50883]. JZ168521–JZ168537 refer to GenBank accession numbers of transcripts from *T. bicarinatum* contig recovered from our previous study [13]. Amino acids that are identical in all or the majority of the sequences are highlighted with different shades of blue that represent their degree of conservation. The active peptide sequence of bicarinalin identified in a previous study [21] is indicated by a red box. Putative signal peptide is underlined.

Members of the pilosulin-like peptides were also identified in our library. The status and features of these peptides have been discussed in our previous work where we showed that different isoforms and families of pilosulin-like sequences occur in *T. bicarinatum*[13]. Recently, two short peptides (named bicarinalin and peptide2) from *T. bicarinatum* venom have been characterized by *de novo* sequencing using mass spectrometry and Edman degradation [21, 22]. The authors show that these mature peptides, identified among the most abundant in the venom of this ant species have no homolog in the public database. In our study, we have recovered these peptides and identified their cDNA sequences (Additional file 2: Table S1). However, when we blast the whole peptide sequences predicted from the cDNA, we found that their leader sequence and propeptide have homologies with those of pilosulin sequences (Figure 4). Moreover, the bicarinalin has been shown to have a potent and broad antibacterial activity as much as pilosulins [21]. Hence, it seems that the bicarinalin and peptide2 or the pilosulin-like forms from *T. bicarinatum* have the same features as some bioactive peptides in that they have a conserved secretory leader and propeptide sequence but differ greatly in their mature peptide [23, 24]. This fact has been attributed to recombination as a toxin diversity-generating mechanism giving individualistic utilization of specific isoforms [25].

At a lower frequency, dipeptidylpeptidase IV has also been detected among the ‘over-expressed’ contig group (1% of the venom toxins, Figure 3). This enzyme is widely distributed in animal tissues and has a highly glycosylated serine protease that cleaves N-terminal dipeptides [6]. A role in the processing of major venom compounds is ascribed to this protein [26].

In addition to the toxin found in the ‘over-expressed’ transcripts, possible toxins and related venom compounds were identified in this work among relatively less expressed transcripts (Additional file 2: Table S1). As some of these toxins/toxins-like have not been previously described from ant species and/or are potential pharmacological targets, a comparative study with families of related venom proteins has been investigated and discussed in respect to their amino-acid features.

### Venom allergen Sol II/ IV-like

**Figure 5:**
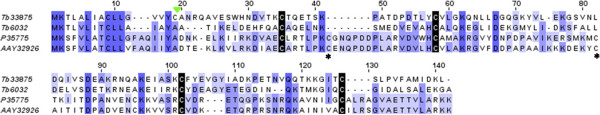
**Amino acid sequence alignment of Sol II and Sol II- like from different ant species.** The sequences of *Sol* II- like from *T. bicarinatum* Tb33875 and Tb6032 were aligned with *Sol i* II from *Solenopsis invicta* [GenBank: 549179] and *Sol g* II from *Solenopsis geminita* [GenBank: 63099693]. Strictly conserved cysteines are indicated by black shading. Cysteines that are unique to *Solenopsis* sequences are indicated by asterisks. The predicted signal peptide cleavage site for *T. bicarinatum Sol* II-like is indicated by a solid green triangle.

Interestingly, members of the venom allergen *Sol* II and the *Sol* IV have been identified in our study. Two contigs Tb6032 and Tb33875 (478 and 616 bp, respectively) have shown similarities with *Sol* II allergen described in some fire ant species. The predicted *Sol* II-like amino-acid sequences are 108 amino-acid long for Tb6032 and 107 for Tb33875 with a putative predicted signal peptide cleavage site for both sequences (Figure 5). The Tb6032 showed a significant identity (32%) with the queen venom protein *Sol g* II precursor of the species *Solenopsis geminata* and 32% identity for the *Sol i* II allergen of the species *S. invicta*. The Tb33875 showed 23% and 26% identity with these sequences, respectively. The amino-acid alignment shows a consistent conservation of the cysteine patterns and a significant conservation of the predicted signal sequence (Figure 5) which suggests the presence of *Sol* II venom allergen in *T. bicarinatum*. This finding is interesting given that up to now these venom allergens have never been identified in other animal venoms and are regarded to be specific to some *Solenopsis* species [7, 27]. The main features distinguishing *T. bicarinatum Sol* II-like from its *Solenopsis Sol* II counterpart are the number of cysteine residues (4 against a number of 7 cysteines for *Sol i* II) and the deletion of seven amino acid residues near the N-terminal and 4 in the C-terminal part.

**Figure 6:**
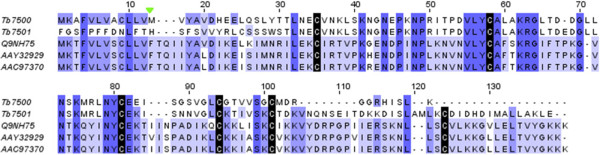
**Amino acid sequence alignment of** ***Sol*** **IV and** ***Sol*** **IV-like from different ant species.** The sequences of *Sol* IV-like from *T. bicarinatum* (Tb7500 and Tb7501) were aligned with *S. invicta Sol i* VI from worker [GenBank: AAC97370] and queen [GenBank: AAY32929] and *Sol g* IV from *S. geminita* [GenBank: Q9NH75]. Strictly conserved cysteines are indicated by black shading. The predicted signal peptide cleavage site for *T. bicarinatum Sol* II-like is indicated by a solid green triangle.

In the same way, two transcripts of *T. bicarinatum* (Tb7500 and Tb7051) have been found similar to the venom allergen *Sol* IV, with consistent identity of 30 to 41%. The two contigs from *T. bicarinatum* are found more related to each other (65% of identity) than to *Sol* IV from the fire ant species. Given that the cysteine pattern is completely conserved along with three amino-acid motifs of more than 3 residues (Figure 6), this suggests that *Sol* IV-like venom allergens occur in *T. bicarinatum* venom. As for the *Sol* II, *Sol* IV is exclusively described from ants of the genus *Solenopsis* and has never been identified in any other insect venom [7, 27].

*Sol* II and *Sol* IV have been described as related to each other in their amino-acid sequences but different in their expression: whereas *Sol* II occupy the two thirds of the protein venoms with important allergen reactivity, *Sol* IV represent 5 to 9% of the protein venoms with less common patient reactivity [27]. In our library, the predicted *Sol* IV-like has not been recorded among the ‘over-expressed’ transcripts, unlike venom allergens 3 and 5, but has showed higher expression rate than *Sol* II-like (Additional file 2: Table S1), mirroring a possible different venom dynamic expressions across ant species. Recently, a functional analysis of the *Sol* II from the species *S. invicta* emphasizes that this protein may play a role in capturing and/or transporting small hydrophobic ligands such as pheromones, odors, fatty acids, or short-living hydrophobic primers [28]. This is interesting in that some detected odor binding proteins in this study (Additional file 2: Table S1) and in our previous study [13] might be investigated as possible venom allergens [29].

### T. bicarinatum PLA2-like

**Figure 7:**
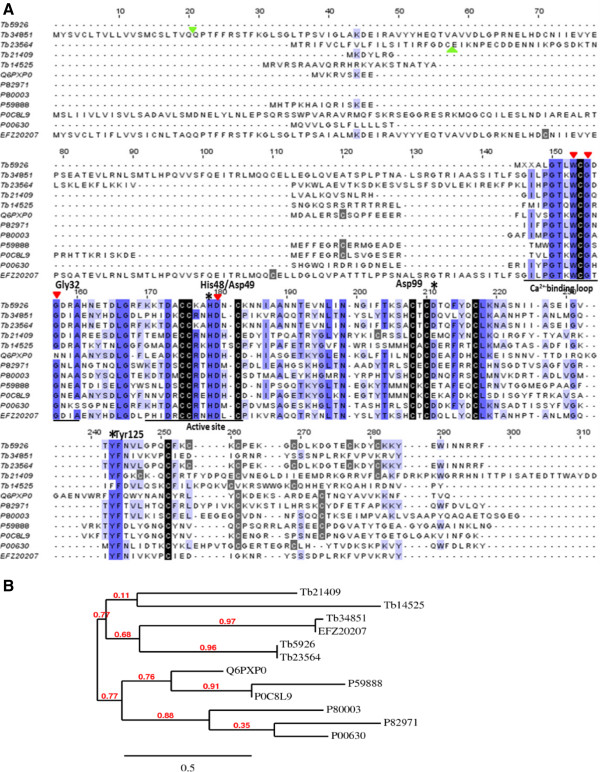
**The predicted phospholipase A2-like (PLA2) from** ***T. bicarinatum*** **. (A)** Amino acid alignment of the sequences of PLA2-like from *T. bicarinatum* (Tb21409, Tb14525, Tb34851, Tb5926 and Tb23564) with PLA2 from *Apis mellifera* [GenBank: P00630], *Bombus terrestriris* [GenBank: P82971], *S. invicta* [GenBank: EFZ20207], *Heloderma suspectum* [GenBank: P80003], *Hadrurus gertschi* [GenBank: P0C8L9], *Pandinus imperator* [GenBank: P59888] and *Anuroctonus phaiodactylus* [GenBank: Q6PXP0]. Strictly conserved and less conserved cysteines are indicated by black and grey shading, respectively and the predicted signal peptide cleavage sites for *T. bicarinatum* PLA2-like is indicated by a solid green triangle. The underlined regions indicate the Ca^2+^ binding loop and the active site: solid red triangle, Ca^2+^ binding residues; *, active site residues. The numbering of the amino acid (Gly32, His48, Asp49 and Asp99) follows that of the PLA2 from bovine pancreas [31]. **(B)** Phylogenetic relationships of PLA2 from venomous animals based on amino acid sequence alignment. Phylogeny has been performed using the maximum likelihood method implemented in the PhyML program at http://www.phylogeny.fr[72]. Numbers at nodes indicate bootstrap supports based on 100 replicates. Protein codes are as described in Figure 7A.

Phospholipases (PLAs) are relatively common in social Hymenoptera venoms occurring in different forms and are able to disrupt the phospholipid layers of several types of biological membranes responsible of many hemolytic and neurotoxic effects (*eg*: [30]). In this work, five different contigs from *T. bicarinatum* matched with databases proteins of phospholipases A2 (PLA2) from Hymenoptera organisms but also from lizard and scorpion species (Figure 7A). One contig (Tb34851) was found to be closely related (91.29% of similarities) with PLA2 previously described in the fire ant *S. invicta*. The remaining four contigs (Tb14525, Tb23564, Tb5926 and Tb21409) showed a mean range similarities of 31% for *S. invicta* and 33% for *Apis mellifera*. According to the amino-acid alignment, *T. bicarinatum* PLA2-like sequences show a high conservation of cysteine patterns and are relatively well conserved in previously identified Ca^2+^–binding loop region and active sites (Figure 7). The highly conserved active site residue His48 and the Ca^2+^–binding residues Gly32 and Asp49, available in group III PLA2 amino acid sequences [31] are also well conserved in *T. bicarinatum*. Taken together with the phylogenetic analysis (Figure 7B), we could assume that these PLA2-like from *T. bicarinatum* belong to the Group III family that have been identified in bee, lizard and scorpion venoms and characterized by low molecular mass (13–18 kDa) and Ca^2+^-dependent enzyme activities [32]. It is worth to notice however a substitution of the highly conserved active site residue Tyr125 with a phenylalanine in the contig Tb14525 of *T. bicarinatum*. As substitution in the active site and/or binding residues is critical and could result in inactive PLA2 [33], this substitution may affect the catalytic activity of this PLA2 *T. bicarinatum* form. In this work, different PLA2-like contigs were identified and the maximum-likelihood phylogeny tree supports the existence of different *T. bicarinatum* PLA2-like clusters that show a closer relationship with PLA2 from *S. invicta* than with the other aligned species from lizard and scorpion (Figure 7B). The occurrence of different PLA2 forms is in agreement with previous studies suggesting that this enzyme occurs as a series of different isoforms and/or post-translationally modified forms [34].

PLA2 was previously reported in social Hymenoptera as highly hemolytic. Its allergenic effect however was reserved to honey bee and wasp rather than ants [35]. A previous study on Hymenoptera venom PLA2 activities (including species of bee, wasp and ant) showed that each of the studied venom PLA2 exhibits lineage related specific pattern and that the chemical composition of hymenopteran venoms is biologically linked to the behavior and biology of the producing organism [36]. Indeed, a comparative study including 9 species from wasps and 9 from ants showed that phospholipase activities have generally higher levels among wasps than ants and even among ant species, differences in phospholipase concentration were also reported. While the enzyme-rich venom of the harvester ant (*Pogonomyrmex badius*) was reported to contain high concentrations of phospholipase A2 [37], that from the ant *Myrmica ruginodis* seems to lack detectable amounts of these enzyme activities [12]. In our study, PLA2-like transcripts from *T. bicarinatum* venom were not detected among the ‘over-expressed’ contigs suggesting a low enzyme activity. Previous study on a closer species (*Tetramorium caespitum*) has shown that no activity from this enzyme was detected [12], making evidence of the existence of different patterns of venom expression among and across taxa in Hymenoptera even though these venoms share a degree of similarity in activity [36].

### T. bicarinatum PLA1-like

**Figure 8:**
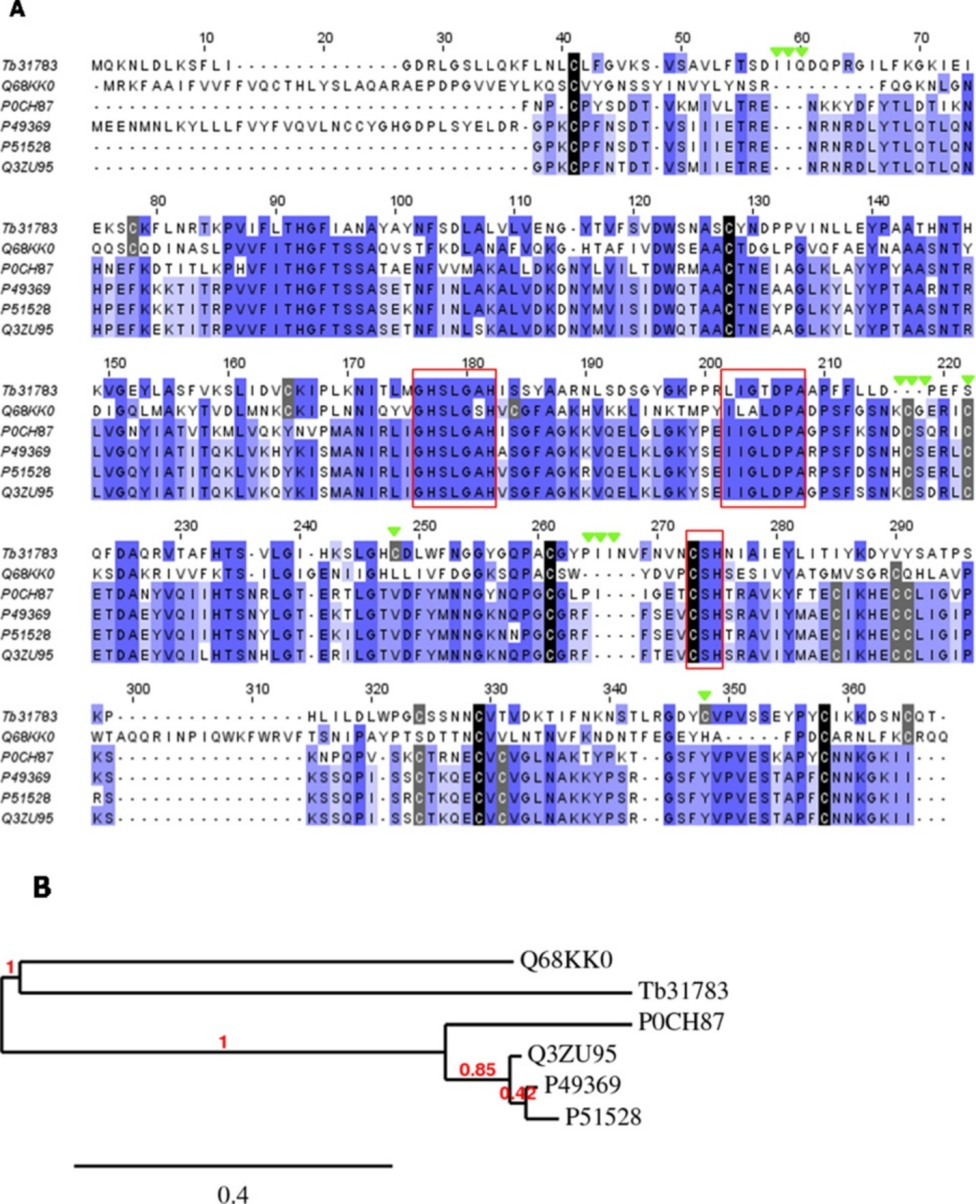
**The predicted phospholipase A1-like (PLA1) from** ***T. bicarinatum*** **. (A)** Amino acid alignment of the sequences of PLA1-like from *T. bicarinatum* Tb31783 with PLA1 from *S. invicta* [GenBank: Q68KK0], *Vespa crabro* [GenBank: P0CH87], *Vespula vulgaris* [GenBank: P49369], *Vespula maculifrons* [GenBank: P51528] and *Vespula germanica* [GenBank: Q3ZU95]. Red boxes indicate PLA1 active sites according to [38] and green arrowheads indicate unique features for *T. bicarinatum* PLA1-like. Strictly conserved and non-conserved cysteine residues are shaded with black and grey, respectively. **(B)** Phylogenetic relationships of PLA1 from insect species based on amino acid sequence alignment. Numbers at nodes indicate bootstrap supports based on 100 replicates. Protein codes are as described in Figure 8A.

One 1284 bp long contig (Tb31783) from *T. bicarinatum* has been identified as venom phospholipase A1 (PLA1) and presented homologies with PLA1 of different Hymenoptera species. Figure 8 shows the amino-acid alignment of the predicted 330 amino acid sequence from this contig with related vespid venom phospholipases and the *Sol i* 1 from *S. invicta*. Identity matrix shows a similarity of 31% with *Sol i* 1 from *S. invicta*, 36% with PLA1 from *Vespa crabro*, 34% for *Vespula germanica*, 34% for *Vespula maculifrons* and 35% for *Vespula vulgaris*. The sequence alignment shows conservation of the regions that form the enzyme active site and variation in the outer regions as described in [38]. The cysteine pattern displayed by *T. bicarinatum* represents unique features comparing to the other PLA1 sequences. For example, the conserved cysteine residues at positions 217 and 222 are replaced by a deletion and a serine insertion, respectively. The missing cysteine seems to occur at the position 248 (Figure 8A). In addition, two tri-amino acid insertions and one tri-amino acid deletion occur at positions 58–60, 265–267 and 216–218, respectively. According to the phylogeny analysis, *T. bicarinatum* PLA1-like follows the Hymenoptera phylogeny and appears to be most closely related to the fire ant phospholipase than to vespid clusters with well supported bootstrap values (Figure 8B). This relatedness is in part due to the conserved cysteine residues between both species at positions 78, 165 and 365 (Figure 8A). However, the insertion of 12 residues near the C-terminal previously described in this fire ant was not detected for *T. bicarinatum*[38]. The PLA1 has been described in several Hymenoptera venoms and it has been shown to have no sequence similarity with other known phospholipases, but has sequence similarity with mammalian lipases [39]. Its occurrence in Hymenoptera venoms was mainly associated with tissue damages and venom diffusion [29, 34].

According to the differential expression analyses of transcripts abundance, it is likely that the PLA1-like and PLA2-like identified in *T. bicarinatum* venom are not among the major allergens and venom components in this ant species. However, providing ‘new’ sequence features from *T. bicarinatum* venom of these enzymes could be of great importance, especially to help studies aiming to build more complete microarrays of proteins for allergy diagnosis. In addition, studies of these enzymes (especially PLA2) become useful tools for understanding ion channel structure and activity. The use of synthetic analogues from these toxins is suggested to lead to the development of new therapeutic agents and strategies for treatment of ion channel-related diseases [40]. Besides, PLA2 have been intensively described from snake venoms as antitumor targets (*eg*[41]) and recently it was suggested that PLA2 from honey bee venom should be tested for the preparation of cell-based cancer vaccines [42].

#### Hyaluronidase

**Figure 9:**
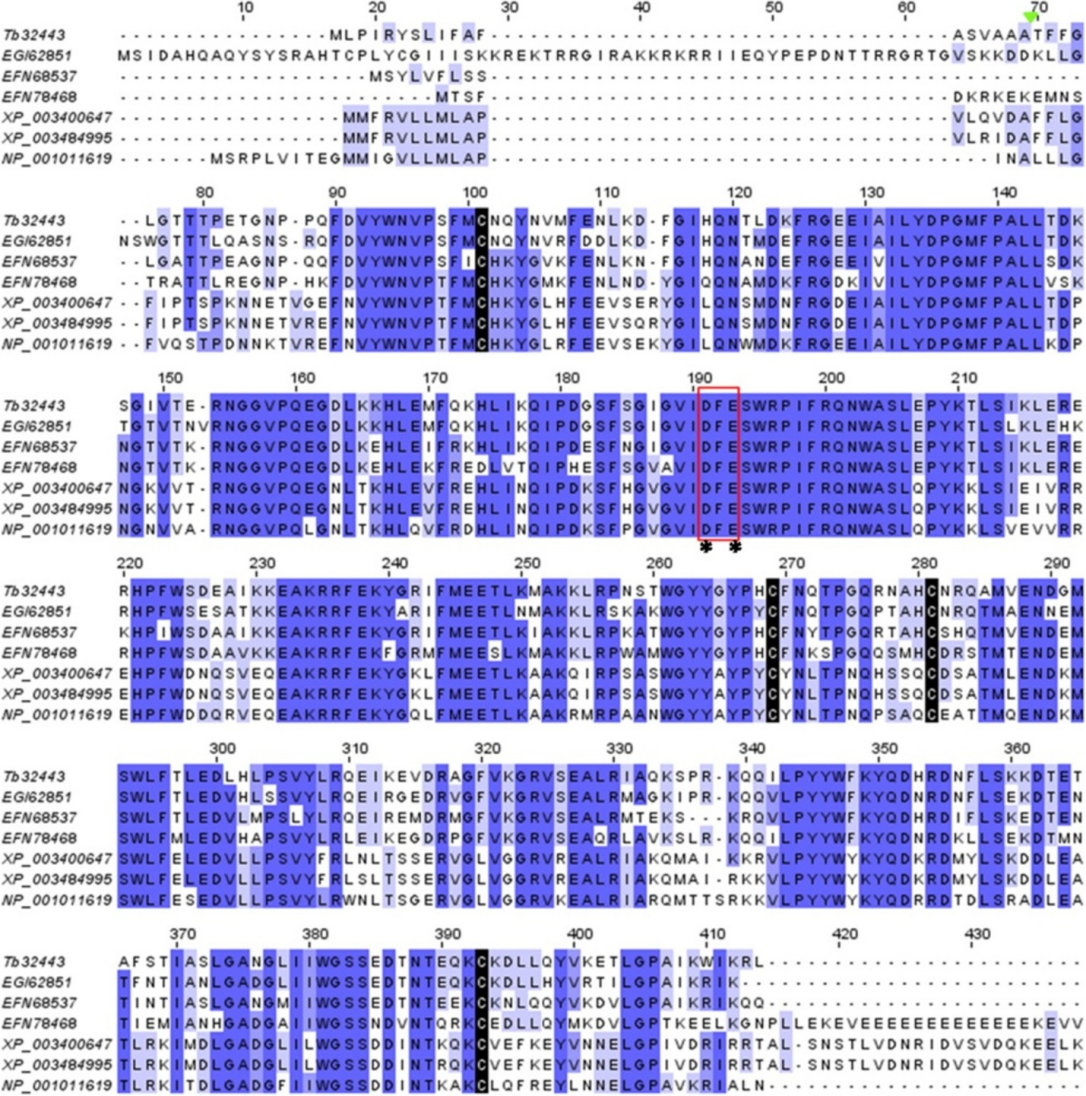
**Amino acid sequence alignment of hyaluronidases from insect species.** The deduced hyaluronidase from *T. bicarinatum* Tb Tb32443 was aligned with hyaluronidases from *A. mellifera* [GenBank: NP_001011619], *Acromyrmex echinatior* [GenBank: EGI62851], *Camponotus floridanus* [GenBank: EFN68537], *Harpegnathos saltator* [GenBank: EFN78468], *Bombus terrestris* [GenBank: XP_003400647] and *Bombus impatiens* [GenBank: XP_003484995]. The tri amino acid residues –DFE– is highlighted by a red rectangle and the active site residues by asterisks. Strictly conserved cysteines are indicated with black shading and the predicted signal peptide cleavage site is indicated by a solid green triangle.

A 1640 bp cDNA sequence (contig Tb32443) composed of 384 amino acid full-length ORF was identified as hyaluronidase in *T. bicarinatum* venom. The putative amino acid sequence showed a relative high similarity to hyaluronidase sequences from other ant species (a mean range similarity of 75%) and to *A. mellifera* (56%) and *bombus sp*. (57%). Structural features described in venom hyaluronidases (HYALs) were highly conserved in *T. bicarinatum,* namely the four cysteine residues forming the two disulfide bridges and the active site residues (Figure 9). The –DFE- motif known to be extremely conserved and present in the active site of all of these molecules was also conserved [43] suggesting that the contig Tb32443 from *T. bicarinatum* encodes HYAL enzymes. HYALs are in general among the more conserved hymenopteran allergens compared to other toxins such as the venom allergen 5 and the PLA1 [43, 44]. They were described to act as a ‘venom spreading factor’ in scorpion, snake, bee, and wasp venoms by hydrolyzing hyaluronic acid, a major component of the interstitial barrier, hence increasing the tissue permeability and easing venom component diffusion [17, 45]. The expression of HYALs in Hymenoptera is very different depending on the organism. They were found in high level in the honey bee and some wasps but as a minor venom allergen in other organisms like the vespid yellow jacket [44]. In ants, enzymes with hyaluronidase activity are not described as a major component except for the Harvester ant *Pogonomyrmex*[17, 46]. In our study, the generated sequences matching HYALs were about 4 times more expressed in the venom gland sequenced sample than in the ant body (Additional file 2: Table S1). Given that the venom gland library was amplified prior to sequencing, only a biochemical characterization and/or a quantitative PCR should elucidate its actual expression level in *T. bicarinatum*.

#### Venom serine proteases

**Figure 10:**
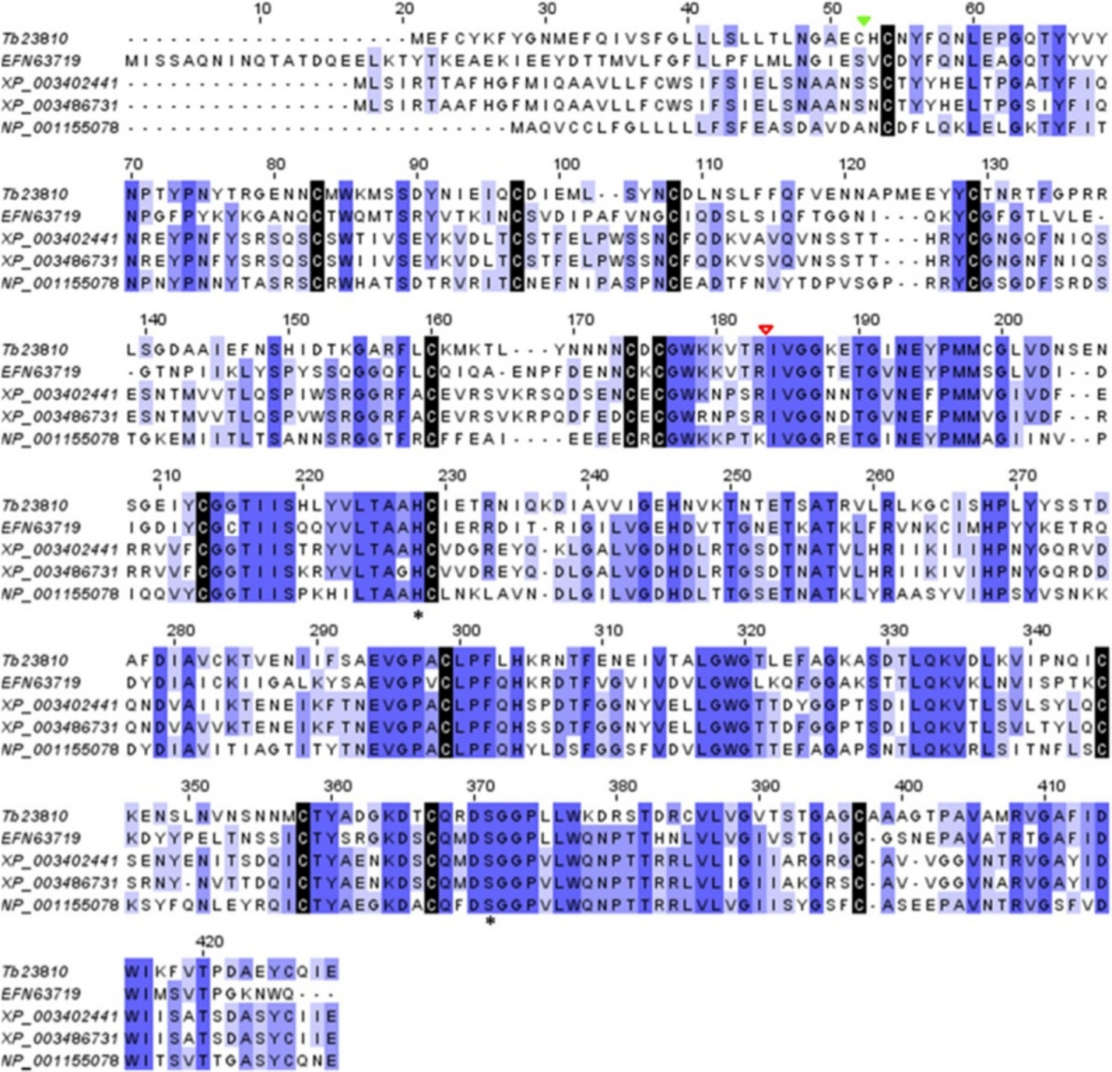
**Amino acid sequence alignment of venom serine protease (VSP) from Hymenopteran species.** The predicted VSP from *T. bicarinatum* TB23810 was aligned with *Camponotus floridanus* [GenBnank: EFN63719], *Bombus terrestris* [GenBnank: XP_003402441], *Bombus impatiens* [GenBnank: XP_003486731] and *Nasonia vitripennis* [GenBnank: NP_001155078]. Strictly conserved cysteines are indicated with black shading. The conserved residues in the catalytic triad of the Serine Protease domain [His (H), Asp (D), and Ser (S)] are indicated with asterisks and the predicted cleavage site of the catalytic serine protease domain with an open red triangle according to [47].The cleavage site of the predicted signal sequence is indicated by a solid green triangle.

Two transcripts of the contig Tb23810 of 1494 bp were identified to encode Venom Serine Protesaes (VSPs). The predicted *T. bicarinatum* VSPs have matched with VSPs from Hymenoptera species, namely of the ant species *Camponotus floridanus* with 49% identity. 41% and 40% of identity have been recorded for the bumlbee species *Bombus terrestris* and *Bombus impatiens*, respectively and 41% for the wasp *Nasonia vitripennis*. Consistent similarities with previously described VSP features were conserved, including the catalytic triad (Ser, His, and Asp) (Figure 10), that represents the main criterion for classification of a protein as a serine protease [47]. Serine proteases are multifunctional enzymes that play important roles in the immune response and hemostasis and are considered important allergens with significant IgE binding activity [48]. Recently, VSP from the bumblebee species *Bombus ignites* venom was identified as a prophenoloxidase-activating factor in insects, triggering the phenoloxidase cascade and inducing thereby a lethal melanization response in target insects; whereas in mammals, it was shown to act similarly to snake VSP, which exhibits fibrinogenolytic activity [47]. Like HYALs, VSPs were suggested to act as ‘spreading factor’ improving the diffusion of venom components and are likely involved in the process of their maturation or activation [49].

#### Non hymenopteran toxins

**Figure 11:**
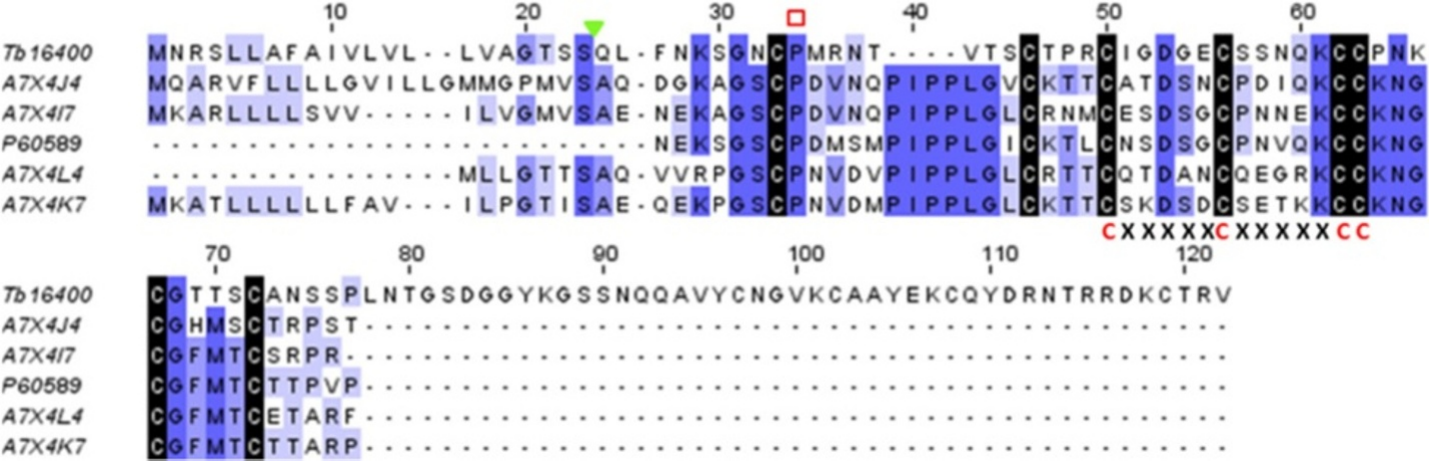
**Amino acid sequence of venom waprins from** ***T. bicarinatum*** **and snake species.** The waprin-like sequence from *T. bicarinatum* Tb16400 was aligned with waprin snake venoms of the species *Rhabdophis tigrinus tigrinus* [GenBank: A7X4J4], *Thrasops jacksonii* [GenBank: A7X4I7], *Naja nigricollis* [GenBank: P60589], *Liophis poecilogyrus* [GenBank: A7X4L4] and *Philodryas olfersii* [GenBank: A7X4K7]. The conserved cysteines are shaded with black and the prolyl residue described in the WAP family domain is indicated by an open red rectangle. The signature pattern of cysteine residues (CxxxxxCxxxxxCC) in the central region found in all WAP-motif proteins is indicated. The signal peptide of the predicted *T. bicarinatum* waprin-like Tb16400 is indicated by a solid green triangle.

The blast search against nr database of *T. bicarinatum* contigs Tb16400 and Tb34742 of 1303 bp and 1169 bp, respectively showed similarities with waprin-like (WAPs) proteins from Hymenoptera species that were submitted to GenBank following whole genome sequencing or genome mining. In order to check the possible homologies with known WAP from other venomous species, a blast search against an in-house toxin database (see Methods section) has been achieved and has revealed homologies with snake-venom WAP. An average similarity of 33% with the matched snake species was recorded. Domain search blast of the identified contigs against the PROSITE database [50] revealed that the predicted sequences (94 amino acid long each) contain the WAP-type ‘four-disulfide core’ domain profile or whey acidic protein motif. In addition, the signature pattern of cysteine residues (CxxxxxCxxxxxCC) in the central region found in all WAP-motif proteins [51] was also identified (Figure 11), which suggests that snake-venom WAP-like are possibly expressed in *T. bicarinatum* venom glands. To the best of our knowledge, WAPs have never been reported from Hymenoptera venoms. The WAP family has been described from many tissue types and organisms and has been found to have many functions, including, immune-modulation, anti-protease and anti-bacterial activities [52, 53]. Recently, they have been described from snake venoms and from amphibian skins, each time associated with anti-microbial activities [51, 54–56]. The identification of WAP from Hymenoptera venom and precisely from ant venom in this study could be of great interest regarding the vast array of its biological functions and should stimulate the investigation of their activity against a panel of micro-organisms.

**Figure 12:**
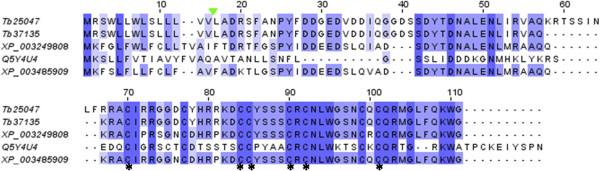
**Amino acid sequence of agatoxin from arthropod species.** The agatoxin-like sequences from *T. bicarinatum* Tb37135 and Tb25047 were aligned with *Bombus impatiens* [GenBank: XP_003485909], *Apis mellifera* [GenBank: XP_003249808] and the spider agatoxin of the species *Agelena orientalis* [GenBank: Q5Y4U4]. Conserved cysteine residues are indicated by asterisks and the cleavage site of the predicted signal sequences from *T. bicarinatum* is indicated by a solid green triangle.

In addition to WAPs-like discussed above, agatoxin-like sequences have also been detected in our analysis (Additional file 2: Table S1). Blast search against specific toxin in-house library shows a similarity of 28% and 29% of the contigs Tb37135 and Tb25047, respectively, to agatoxin sequence from the spider *Agelena orientalis* [GenBank accession: Q5Y4U4.1], with a completely cysteine pattern conservation (Figure 12). The amino acid sequences deduced from both contigs have an agatoxin signature of 16 residues N-terminal signal peptide and 46 predicted mature residues. NCBI Blast of these two contigs shows consistent similarities (ranging from 68 to 91%) with predicted agatoxin-like isoforms from the honey bee *A. mellifera* [GenBank accession: XP_003249808] and the bumble bee *Bombus impatiens* [GenBank accession: XP_003485909]. These agatoxin-like have been predicted from an automated computational analysis rather than validated from venom gland tissues. Recently, based on a combined cDNA cloning and shotgun deep sequencing approach, the spider U8 agatoxin has been recovered from the venom gland of the ant species *D. quadriceps* belonging to the Ponerinae subfamily [8]. As for *T. bicarinatum* the agatoxin sequences are among the minor toxin-related components. This finding may be very useful especially for therapeutics based on new strategies for treatment of ion channel-related diseases. Indeed, agatoxins are high specific ligands for voltage-gated ion channels and are among the potential target neurotoxins that are useful tools for studying channel structure and activity [40].

#### Other toxins

Other putative toxins/enzymes have been detected to be expressed by *T. bicarinatum* venom glands, namely secapin, venom serine carboxylesterase, venom serine carboxypeptidase, lectizyme, disintegrin and metalloproteinase-like, chymotrypsin-like inhibitors. Details on their respective contigs, expression level are provided in (Additional file 2: Table S1). All these predicted toxin-like showed consistent similarities and homology with hymenopteran venom sequences mainly from ant species that have been submitted to public databases in the frame of whole genome sequencing. Their description in this study strengthens their existence/expression in the venom glands.

### No hits and putative ‘new’ venom toxins from *T. bicarinatum*

In addition to the venom peptides and proteins described above, the venom gland library contained about 40% of contigs that were not identified in the public or toxin specific databases (Additional file 3: Table S2). About 3% of these contigs with no match have been found among the arbitrary ‘overexpressed’ contigs where they form the most abundant group (77%). That ‘no-hits’ level reflects and emphasizes the limited amount of information available for *Tetramorium* species and more broadly from other ants in databases. Definitely, this set of “no hit sequences” constitutes a potentially rich reservoir for the identification of novel toxins. In order to investigate the potential of these peptides as ‘new’ toxins, we searched for clues, such as the prediction of signal peptide and cysteine pattern. Details on statistic features of the ‘no-hit’ contigs are provided in (Additional file 3: Table S2). Thus, about 17% of these contigs belonging to the ‘over-expressed’ group have a predicted signal peptide and at least two cysteins. About 8% have signal sequences with no cysteine residues. As peptides from ant venom are more and more suggested to exhibit a defensive role against microbial pathogens, associated with prey introductions and/or ingestion [57], special investigation of *T. bicarinatum* ‘no-hits’ sequences was carried out using the blast search against the antimicrobial peptide database (APD). In addition, their signal peptide sequences were blasted against an in-house toxin-specific signal peptide database. Six original toxin candidates were recorded based on their relative high expression level, presence of signal peptide and on homologies (ranged between 27 to 53%) with established antimicrobial peptides. The length of the predicted mature peptide from these sequences ranged from 23 to 72 amino acids. The features of these putative novel toxins and the amino-acid alignment with their homologs from the APD are provided in (Additional file 4: Table S3). Signal peptide blast result against the in-house toxin signal peptide was not significant except for the contig Tb7117 that shows supported homology (2^e-05^) with pilosulin allergens (Additional file 5: Table S4).

The identified homologs from APD are antimicrobial peptides from the venom secretion of some scorpion and spiders species or from frog skin secretions. The major expressed contig, namely the Tb34031 matches with the Charybdotoxin, an antimicrobial peptide from the yellow scorpion *Leiurus quinquestriatus hebraeus* with a sequence similarity of 27.94% (Additional file 4: Table S3 and Additional file 5: Table S4, respectively). Among the 4 cysteine residues that are present in the *T. bicarinatum* peptide, two are conserved with that of the venom scorpion peptide known to target K^+^ channels [58]. The most important sequence homologies were recorded for the contig Tb7101 that matched with 53.57% of similarity with the ponericin W4, an antibacterial and insecticidal peptide from the venom of the ant *Pachycondyla goeldii*[57].

Given the homologies exhibited by the six *T. bicarinatum* predicted peptides identified in our study with antimicrobial peptides and their toxin-like features, it would be very interesting to characterize these peptides with proteomic approaches and test their antimicrobial potential against a panel of micro-organisms.

### Sequence accession numbers

The original, unmerged sequencing reads of the venom gland library were submitted to the National Center for Biotechnology Information (NCBI) Sequence Read Archive under accession number SRR1106145. The processed and assembled data (toxin and nontoxin sequences) investigated in this study were submitted to the GenBank Transcriptome Shotgun Assembly (TSA) database. The TSA project has been deposited at DDBJ/EMBL/GenBank under the accession GASM00000000. The version described in this paper is the first version, GASM01000000.

## Conclusions

In the present study, we generated comprehensive transcriptomic data based on *de novo* assembly from a venom gland transcriptome of the non-model ant species *T. bicarinatum* (Hymenoptera: Formicidae). Given the limited available data from venom of this zoological group, our work greatly expanded the current knowledge of these venoms. In addition, the venom protein diversity and the presence of atypical possible venom peptides recovered in this work indicate that ant venoms are a rather complex pool with currently unknown types of venom peptides that remain to be characterized and would provide a rich unexplored resource for biomedical applications. Furthermore, data generated from sequencing of the whole ant body at transcription level from an ant species could be useful for entomologists.

## Methods

### Ants and rearing conditions

Polygynous colonies of the species *T. bicarinatum* were collected from Brazil (Itabuna, Bahia). The rearing conditions of the ant colonies and venom gland dissection are as described in [13]. Three hundred ant workers were sacrificed and their venom gland pooled in the same sample. They were immediately flash frozen in liquid nitrogen and stored at −80°C until processed. In order to investigate differentially expressed genes, the remaining bodies of the dissected ant workers were pooled in a separate tube and immediately stored in liquid nitrogen, with prior retrieval of stomach and alimentary canal, in order to avoid contamination with genes from micro-organisms which may be present.

### RNA extraction and library preparation

Total RNAs (tRNAs) from venom glands sample were isolated with RNeasy Micro Kit (Qiagen, France) including an on column DNase digestion whereas total RNAs from ant body carcasses were extracted using 400 μl of TRI reagent (Sigma) according to the manufacturer’s protocol. Sequencing and cDNA library preparation were performed by Beckman Coulter Genomics services (http://www.beckmangenomics.com/). Given the very limited amount of the total RNA extracted from the venom glands (7 ng/μl), mRNA from this sample (sample G) was transcribed into cDNA and amplified using the Ovation RNA-Seq System V2 kit, especially applied to limited biological material (NuGEN Technologies Inc.). After cDNA fragmentation, end-repair and purification with the Agencourt® AMPure® XP kit (Agencourt Bioscience, Beckman Coulter, San Carlos, CA, USA), TruSeq sequencing adapters (Illumina) were ligated to the cDNA fragments. Finally, the library was PCR-amplified (14 cycles) to about 20–30 ng/μl using a high fidelity DNA polymerase. For the total RNA sample from ant carcasses (Sample F), poly (A) RNA was isolated and fragmented. First-strand cDNA synthesis was primed with an N6 randomized primer.

### Illumina sequencing

Illumina TruSeq adapters were ligated to the 5′ and 3′ ends of the cDNA of both samples. The cDNA was finally PCR amplified using a proof reading enzyme (Beckman genomics). For Illumina sequencing, the cDNA samples were fractionated on preparative agarose gels in the size range of 300 – 500 bp. PCR amplification was designed for TruSeq sequencing (using HiSeq2000 technology) according to the instructions of Illumina.

### Transcriptome assembly and analysis

Quality of reads generated by deep sequencing was checked within the ng6 environment using fastQC program (available at http://www.bioinformatics.babraham.ac.uk/projects/fastqc/) and Burrows-Wheeler Aligner tool (BWA) to search for contamination [59]. The transcriptome *de novo* assembly was performed using Velvet/Oases [60]. The first step consisted of nine independent assemblies using different k-mers (k-mers for velveth: 25,31,37,43,49,55,61,65,69; parameters for velvetg: −read_trkg yes -min_contig_lgth 100 -cov_cutoff 4 and parameters for oases: −cov_cutoff 4). The raw transcripts files were filtered to retain only 1% of transcripts per locus with a modified version of a Perl script developed at Brown University (https://sites.google.com/a/brown.edu/bioinformatics-in-biomed/velvet-and-oases-transcriptome). Anti-sense chimeras accidentally produced during the assembly step were cut with a home-made script. Then, independent assemblies were pooled, and duplicate/similar transcripts build by close k-mers were removed by a cd-hit-est [61] step (parameters: −M 0 -d 0 -c 0.98) and merged by a TGICL [62] step (parameters: −l 60 -p 96 -s 100,000). After this assembly process, all input reads were mapped back to the set of transcripts using BWA.

### ORFs prediction and functional annotation

Open Reading Frame (ORFs) were predicted by FrameDP software [63] using a home-made reference database consisting of an enriched SwissProt database [64] with venom peptides from Hymenoptera phylum. Blast search (version 2.2.26+) against nr protein and SwissProt databases was performed for ORFs annotation [65]. In addition, domain annotation was achieved with the standalone version of InterProScan [66]. Under Blast2Go software (version 2.5.0) [67], a final annotation was performed by combining Blast and InterProScan results. Furthermore, contigs with no predicted ORFs were also annotated.

In order to detect putative toxins, reviewed databases from Uniprot annotation program Tox-Prot [68] and home-made mature peptide sequences database were used. Additional in-house scripts and known tools as signalP (version 4.0) [69] have been combined to predict signal peptides.

### Alignment and phylogeny analysis

Multiple sequence amino acid alignments allowing comparative study of families of related venom proteins were performed using Muscle (version 3.7) [70], then manually edited and visualized using Jalview software [71]. Phylogenetical analyses were carried out using maximum likelihood method implemented in the PhyML program at http://www.phylogeny.fr[72].

## Electronic supplementary material

Additional file 1:**Distribution of bacteria community in** ***T. bicarinatum*** **according to their contig number.**(DOCX 108 KB)

Additional file 2: Table S1:**Putative toxins from** ***Tetramorium bicarinatum*** **venoms glands.**(DOCX 19 KB)

Additional file 3:**Statistics and features of ‘No hit’ contigs from** ***T. bicarinatum*** **venom gland library.**(DOCX 15 KB)

Additional file 4:**Putative novel types of venom peptide precursors from** ***T. bicarinatum.***(DOCX 16 KB)

Additional file 5:**Statistical details on the putative novel venom peptides characterized from** ***T. bicarinatum.***(DOCX 12 KB)

## Abbreviations

ESTs: Expressed sequence tags
GO: Gene ontology
PLAs: Phospholipases
PLA2: Phospholipase A2
PLA1: Phospholipase A1
HYALs: Hyaluronidases
VSPs: Venom serine protesaes
WAPs: Waprins
APD: Antimicrobial peptide database
tRNAs: Total RNAs
BWA: Burrows-wheeler aligner
ORFs: Open reading frames.
