# Supplementary figures and images for: De Novo sequencing and transcriptome analysis for Tetramorium bicarinatum: a comprehensive venom gland transcriptome analysis from an ant species
Source: BMC Genomics. 2014 Nov 18;15(1):987. doi: 10.1186/1471-2164-15-987 (PMC4256838; doi:10.1186/1471-2164-15-987)

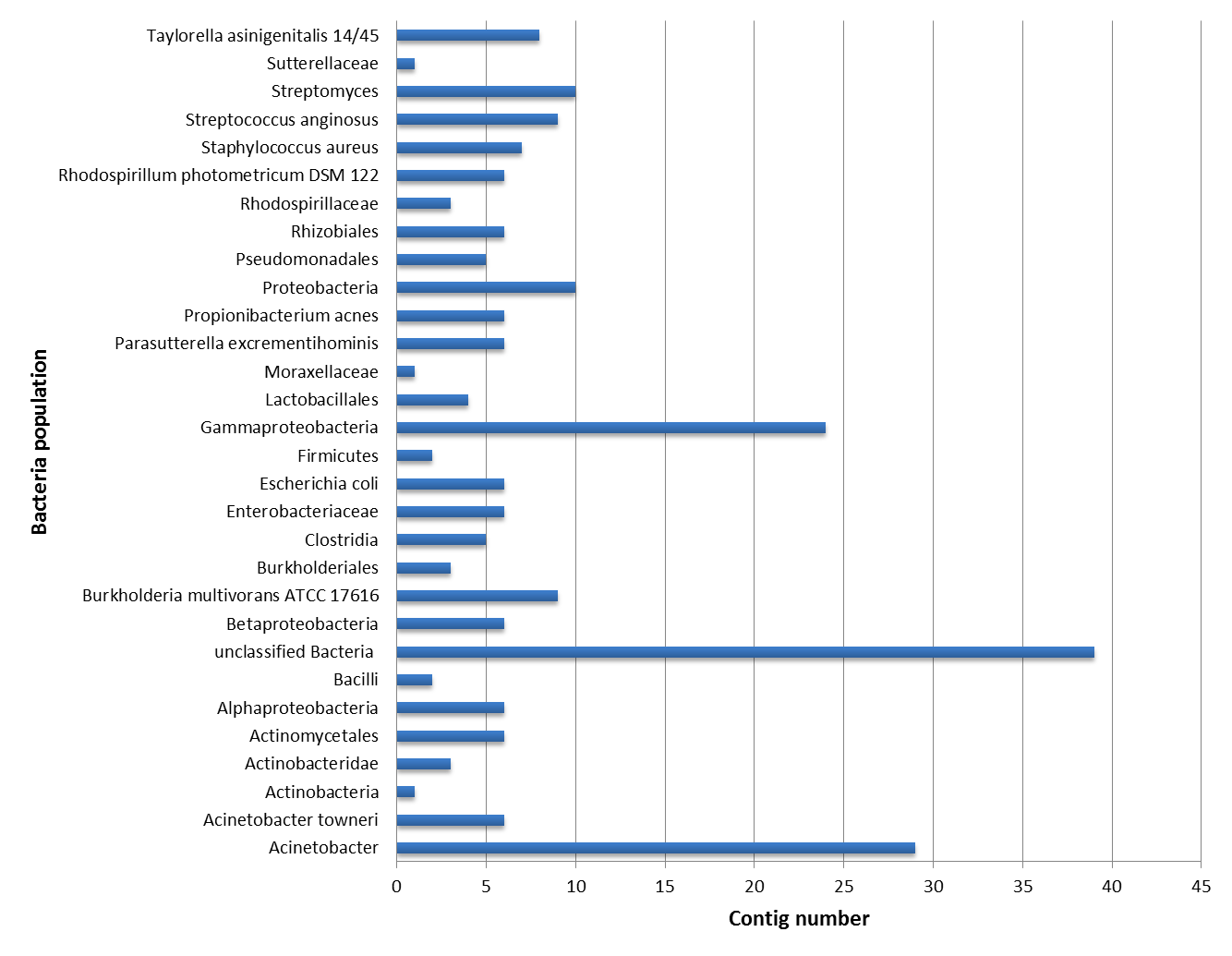

Supplement: Supplementary file 1 — Additional file 1:Distribution of bacteria community in T. bicarinatum according to their contig number.(DOCX 108 KB) [file 12864_2014_6712_MOESM1_ESM.docx]
