# Supplementary material for: De Novo sequencing and transcriptome analysis for Tetramorium bicarinatum: a comprehensive venom gland transcriptome analysis from an ant species
Source: BMC Genomics. 2014 Nov 18;15(1):987. doi: 10.1186/1471-2164-15-987 (PMC4256838; doi:10.1186/1471-2164-15-987)
Supplement: Supplementary file 2 — Additional file 2: Table S1:Putative toxins from Tetramorium bicarinatum venoms glands.(DOCX 19 KB) [file 12864_2014_6712_MOESM2_ESM.docx]

## **Table S1 - Putative toxins from Tetramorium bicarinatum venom glands**

The (*) designates the detection of putative signal peptides, predicted using SignalP 3.0 program [24]. Abbreviations. *PLA2* Phospholipase A2,

*PLA1* Phospholipase A1, *PLD1* Phospholipase D1, *HYAL* Hyaluronidase, *VSP* Venom Serine Protease, *WAP* Waprin, *Ag5*: venom allergen5, *Ag3* venom allergen3, *DPP IV* dipeptidylpeptidase IV, *OR* Odorant receptor, *VCE-6-like*: Venom carboxylesterase 6-like, *VSCP*: Venom serine carboxypeptidase, *Pil 3* pilosulin 3, *Pil 4* pilosulin 4, *Pil* pilosulin

| **Putative**  **identification** | **Cluster name** | **Sequence Length (bp)** | **Total reads in venom gland** | **Total reads**  **in ant body** |
| --- | --- | --- | --- | --- |
| PLA2* | Tb23564 | 3515 | 1047 | 195 |
| PLA2 | Tb5926 | 962 | 568 | 1 |
| PLA2 | Tb14525 | 2416 | 331 | 121 |
| PLA2 | Tb21409 | 1601 | 191 | 14 |
| PLA2* | Tb34851 | 1542 | 383 | 775 |
| PLA1 | Tb31783 | 1284 | 752 | 5 |
| PLA1* | Tb36983 | 1248 | 350 | 20 |
| PLA1* | Tb8280 | 2473 | 839 | 94 |
| PLD1 | Tb23396 | 4815 | 769 | 54 |
| HYAL* | Tb32443 | 1640 | 1387 | 339 |
| Disintegrin | Tb17809 | 1517 | 963 | 29 |
| Disintegrin | Tb2323 | 2642 | 564 | 94 |
| Disintegrin * | Tb13686 | 915 | 105 | 18 |
| VSP | Tb14811 | 2021 | 473 | 136 |
| VSP | Tb21565 | 4142 | 506 | 24 |
| VSP * | Tb23810 | 1494 | 227 | 17 |
| WAP-like* | Tb16400 | 1303 | 1516 | 219 |
| WAP-like* | Tb34742 | 1169 | 1046 | 207 |
| Agatoxin-like* | Tb37135 | 1232 | 881 | 5 |
| Agatoxin-like* | Tb25047 | 1406 | 935 | 4 |
| *Sol* II-like* | Tb6032 | 478 | 516 | 50 |
| *Sol* II- like | Tb33875 | 616 | 695 | 0 |
| *Sol* IV- like* | Tb7500 | 627 | 949 | 6 |
| *Sol* IV- like | Tb7501 | 658 | 869 | 5 |
| Ag 5 | Tb3545 | 1038 | 5048 | 2 |
| Ag 5 | Tb37061 | 203 | 1261 | 1 |
| Ag 5 | Tb3616 | 201 | 1352 | 1 |
| Ag 5 | Tb3596 | 207 | 2796 | 1 |
| Ag 5 | Tb3605 | 223 | 3074 | 2 |
| Ag 5 | Tb3559 | 320 | 5524 | 4 |
| Ag 3* | Tb20838 | 1344 | 1862 | 175 |
| Ag 3 | Tb7796 | 2105 | 3932 | 72 |
| Ag 3 | Tb14947 | 1486 | 2024 | 72 |
| Ag 3 | Tb3533 | 449 | 62834 | 30 |
| Ag 3 | Tb3536 | 876 | 22999 | 21 |
| Ag 3* | Tb3534 | 990 | 1510 | 1 |
| Ag 3* | Tb3538 | 1057 | 1986 | 2 |
| AcP | Tb3652 | 207 | 2985 | 1 |
| AcP | Tb3653 | 370 | 1971 | 1 |
| AcP | Tb36589 | 217 | 1239 | 1 |
| AcP | Tb3660 | 208 | 1931 | 1 |
| AcP | Tb33764 | 222 | 1592 | 1 |
| AcP * | Tb7233 | 3540 | 3209 | 187 |
| DPP IV | Tb429 | 1171 | 6249 | 74 |
| DPP IV | Tb35169 | 1307 | 3978 | 37 |
| DPP IV | Tb36175 | 232 | 1036 | 7 |
| DPP IV * | Tb32846 | 890 | 957 | 163 |
| DPP IV * | Tb33420 | 539 | 62 | 0 |
| OR | Tb3953 | 1598 | 883 | 17 |
| OR | Tb3954 | 985 | 627 | 3 |
| OR | Tb3955 | 1210 | 969 | 16 |
| Protease inhibitor* | Tb30751 | 1351 | 435 | 149 |
| Secapin | Tb10640 | 656 | 1179 | 18 |
| Secapin | Tb10642 | 865 | 1333 | 4 |
| Secapin | Tb10643 | 501 | 1238 | 40 |
| VCE-6-like | Tb35530 | 1001 | 578 | 14 |
| VSCP* | Tb37614 | 1718 | 1006 | 171 |
| VSCP* | Tb20519 | 2043 | 980 | 225 |
| Lectizyme* | Tb25374 | 930 | 189 | 18 |
| Lectizyme* | Tb34526 | 1063 | 1077 | 750 |
| Pil 3-like | Tb10722 | 298 | 39700 | 18 |
| Pil 3-like | Tb7120 | 296 | 10208 | 8 |
| Pil 3-like | Tb32677 | 439 | 112600 | 60 |
| Pil 4-like* | Tb7121 | 341 | 100141 | 37 |
| Pil 4-like* | Tb3631 | 621 | 1367 | 0 |
| Bicarinalin/pil-like | Tb34317 | 202 | 50242 | 11 |
| Peptide2/Pil-like | Tb33937 | 1548 | 579009 | 269 |
| Peptide2/Pil -like | Tb34981 | 899 | 1513 | 5 |
| Peptide2/Pil -like | Tb35781 | 240 | 738385 | 372 |
