# Supplementary material for: De Novo sequencing and transcriptome analysis for Tetramorium bicarinatum: a comprehensive venom gland transcriptome analysis from an ant species
Source: BMC Genomics. 2014 Nov 18;15(1):987. doi: 10.1186/1471-2164-15-987 (PMC4256838; doi:10.1186/1471-2164-15-987)
Supplement: Supplementary file 3 — Additional file 3:Statistics and features of ‘No hit’ contigs from T. bicarinatum venom gland library.(DOCX 15 KB) [file 12864_2014_6712_MOESM3_ESM.docx]

## **Table S2 - Statistics and features of ‘No hit’ contigs from T. bicarinatum venom gland library**

*Number of clusters was defined using CD-hit algorithm [72], Square brackets indicate average number of sequence within a cluster.

|  | Venom gland library | ‘Over-expressed’ group |
| --- | --- | --- |
| Number of contigs that have no hits | 14400 (40%) | 386 (77%) |
| Number of hits with predicted ORF |  |  |
| with signal peptide and at least 2 cysteines | 367 | 65 |
| with signal peptide and without cysteines | 268 | 30 |
| Number of clusters* | 53[2] | 13[2] |
| Number of single sequence | 314 | 360 |
