# Supplementary material for: De Novo sequencing and transcriptome analysis for Tetramorium bicarinatum: a comprehensive venom gland transcriptome analysis from an ant species
Source: BMC Genomics. 2014 Nov 18;15(1):987. doi: 10.1186/1471-2164-15-987 (PMC4256838; doi:10.1186/1471-2164-15-987)
Supplement: Supplementary file 4 — Additional file 4:Putative novel types of venom peptide precursors from T. bicarinatum.(DOCX 16 KB) [file 12864_2014_6712_MOESM4_ESM.docx]

## **Table S3 - Putative novel types of venom peptide precursors from T. bicarinatum**

Numbers between square brackets refer to antimicrobial peptide ID in the Antimicrobial peptide database. Those in red color refer to ‘over-expressed’ contigs from the venom gland of *T. bicarinatum* identified in this study. Cystein residues of *T. bicarinatum* venom predicted peptides are indicated and underlined in red. Details on the antimicrobial peptides that have homologies with predicted peptides from *T. bicarinatum* are as follows:

[AP01503]: **Esculentin-1CPa**; antimicrobial peptide from the skin secretions of the New World frogs *Lithobates capito* (Similarity 33.33%)

[AP00437]: **Charybdotoxin**; antimicrobial peptide from the venom of the yellow scorpion *Leiurus quinquestriatus hebraeus* (Similarity 37.25 %)

[AP00191]: **Gomesin**: Cysteine-rich defense peptide from the spider *Acanthoscurria gomesiana* (Similarity 35%)

[AP00808]: **Hepcidin**; antimicrobial peptide from the bony fish seabream *Sparus aurata* (Similarity 37.93%)

[AP01431]: **Brevinin-2PTe**; antimicrobial peptides from the skin secretions of the Malaysian frogs, *Odorrana hosii* and *Hylarana picturata* (Similarity 31.7%)

[AP00389]: **Ponericin W4**; antibacterial and insecticidal peptides from the venom of the ant *Pachycondyla goeldii* (Similarity 53.57%)

| [Tb10645]  [AP01503] | M I T F L L I S I F I T M V + D G E A T I + I D V P + + I Q C P S G T V K V G N K +  + + + + + + + + + + + + + + F L G + G L I K I + V P A M I + C + A V T + K + + + K C |
| --- | --- |
| [Tb34031]  [AP00437] | M L W L M L Q L I H L L V V V F D R E L F E T Y C T N T T T K + R W I S + C S I S H + + S I G Q A D C + N G R C K W L M R C Y G R C Y G  + + + + + + + + + + + + + + + + + + E + F + T N V S C T T S K E C W + S V C Q R L H N T S R G + + K C M N + + + K + + + K C + + R C Y S |
| [Tb23321]  [AP00191] | C I L F I S L I Q L D F T Y A I G W R C L Q I Y Y Q S C F M Y T M I R  + + + + + + + + + + + + + + + + E C R R L + C Y K Q R C V T Y C R G R |
| [Tb3642]  [AP00808] | M A S A C T W G A Y I I I I T K I M A + K A M A D A M A D A K R C E P D R A R R G L C G L E E R S N Y M F  + + + + + + + + + + + + + + + + + + + + + + + + + + C R F C C R C C P + R + M R G + C G L C C R + + + + F |
| [Tb7117]  [AP01431] | M I I S F L I F I K + I A + N V Q A N T V G I L I N I K N + E I + + I I S I V G + + A C H N L + C Y G N H I R T P S E I R T P S E  + + + + + + + + + + + + + + + + + + + + + G F L D S F K N A M I G V A K S + V G K T A L S T L A C + + + K I + D K S C + + + + + + |
| [Tb7101]  [AP00389] | M L M L L L L A N A L A E A S A G I + V T K L I K K G V K L G L + K + + + M A L G K K +  + + + + + + + + + + + + + + + + G I W G T A L + K W G V K L + L P K L V G M A Q T K K Q |
