# Supplementary material for: De Novo sequencing and transcriptome analysis for Tetramorium bicarinatum: a comprehensive venom gland transcriptome analysis from an ant species
Source: BMC Genomics. 2014 Nov 18;15(1):987. doi: 10.1186/1471-2164-15-987 (PMC4256838; doi:10.1186/1471-2164-15-987)
Supplement: Supplementary file 5 — Additional file 5:Statistical details on the putative novel venom peptides characterized from T. bicarinatum.(DOCX 12 KB) [file 12864_2014_6712_MOESM5_ESM.docx]

## **Table S4 - Statistical details on the putative novel venom peptides characterized from T. bicarinatum**

Comparison of total reads in the ant body and the venom gland is provided. Blast search result against an in-house toxin signal peptide database is provided. Number between brackets refer to the e-values

| **Contig name** | **Venom gland** | **Ant carcasses** | **cDNA length (bp)** | **Predicted mature peptide length (Aa)** | **Signal peptide blast result** |
| --- | --- | --- | --- | --- | --- |
| Tb10645 | 1418 | 0 | 1116 | 28 | Conotoxin precursor (1.9) |
| Tb34031 | 266264 | 63 | 764 | 47 | Potassium channel toxin (0.30) |
| Tb23321 | 2244 | 0 | 6556 | 72 | No hit |
| Tb3642 | 3679 | 1 | 206 | 29 | No hit |
| Tb7117 | 4087 | 3 | 229 | 35 | Pilosulin-3a (2e^-05^) |
| Tb7101 | 36243 | 17 | 410 | 23 | No hit |
